# Early Life History of *Alatina* cf. *moseri* Populations from Australia and Hawaii with Implications for Taxonomy (Cubozoa: Carybdeida, Alatinidae)

**DOI:** 10.1371/journal.pone.0084377

**Published:** 2014-01-15

**Authors:** Teresa Carrette, Ilka Straehler-Pohl, Jamie Seymour

**Affiliations:** 1 School of Marine and Tropical Biology, James Cook University, Cairns, Queensland, Australia; 2 Zoological Institute & Museum, Biocentre Grindel, University of Hamburg, Hamburg, Germany; 3 Centre for Biodiscovery and Molecular Development of Therapeutics, Queensland Tropical Health Alliance; James Cook University, Cairns, Queensland, Australia; University of California, Berkeley, United States of America

## Abstract

The early life stages of the cubomedusa *Alatina* cf. *moseri* from Osprey Reef (North Queensland, Australia) and Waikiki (Oahu, Hawaii) were studied using laboratory-based culturing conditions. Spawning populations from both regions were observed with reliable periodicity allowing polyp cultures from these locations to be collected and established under laboratory conditions. The polyps of this species were successfully reared from spawning adults. Polyps of *Alatina* cf. *moseri* were cultured at temperatures of 23–28°C, developed up to 19 tentacles and reached up to 1.70 mm in height. The balloon-shaped hypostomes possessed 4 well-defined lips. The polyps increased their numbers by means of formation of either sedentary polyp buds or creeping-polyp buds, which attached after 2–3 days. Metamorphosis occurred at temperatures of 25–28°C. Development of polyps and medusae were achieved for the first time within the genus *Alatina* and allowed comparisons of early life history between these and other species of the Carybdeida families. The metamorphosis and young medusa of this genus showed characters that differed distinctly from those noted for other Carybdeida species, but are very similar to the one described from Puerto Rico by Arneson and Cutress in 1976 for *Alatina* sp. (named by them *Carybdea alata*). Based on this evidence, the discrepancies in original specimen descriptions and the previous genetic comparisons, we support the suggestion that the two previously described species of *Alatina* from Australia and Hawaii (*Alatina mordens* and *Alatina moseri*) appear to represent artificial taxonomic units and may in fact be the same as the original *Carybdea alata* species named from Puerto Rico. Further taxonomic studies are desperately needed in order to clarify the various species and description discrepancies that exist within this newly proposed genus.

## Introduction

The early life history has not yet been described for Carybdeida species belonging to the newly formed genus *Alatina* [1] In fact, life cycle knowledge is missing for the majority of the cubozoan species [2] only a small number having descriptions of the early stages of the life cycle [3], [4], [5], [6], [7], [8], [9], [10], [11], [12], [13], [14], [15], [16]. Only one complete life cycle, from spawning medusa through sessile stages to spawning medusa, has been published to date for the Caribbean cubozoan *Tripedalia cystophora* Conant, 1897 [17], [18], [19], [20], [21], [22], [23].

Mass spawning of corals and their link to the lunar cycle is a well-known phenomenon across various parts of the globe [24], [25] but very few such lunar based spawning events have been reported in other cnidarians [2]. The lunar linked periodicity of *Alatina moseri* (Mayer, 1906) (previously *Charybdea moseri* Mayer, 1906 or *Carybdea alata* Reynaud, 1830) in Hawaii has been documented since the late 1980's with their predictable arrival on Waikiki beach being reported as the 8^th^ to 10^th^ days after every full moon [2], [26], [27], [28], aggregations of sexually mature *Alatina* were observed from this site by the first and third author on consecutive monthly cycles from Waikiki Beach in Hawaii on various occasions over the last 10 years leading to the hypothesis of a spawning population. A similar observation of sexually mature medusae with regular lunar periodicity (also 8 to 10 days after a full moon) was noted from *Alatina* populations from 1999 onwards at Osprey Reef, which allowed for reliable collections to be made from both these sites.

The order Carybdeida is under revision and the subject of some debate, however, there are currently five accepted families within this group, which are Alatinidae [1], Carukiidae [29], Carybdeidae[30] , Tamoyidae [31] and Tripedaliidae [32]. Within the Alatinidae family there are several species that are under revision and debate continues as to the validity of the different *Alatina* species [33] in particular and how these relate to the original description of *Carybdea alata* that was described by Reynaud [34] for a species from the Atlantic Ocean.

### Species Identification

The population of *Alatina* sp. used in this study, collected from Osprey Reef, is similar in morphology to the described alatinid *Alatina mordens* also reported from the Great Barrier Reef region [1], however, the specimens reported here have 6 eyes per rhopalium (not 2 or 4) and their statoliths are a distinctly light amber colour and jellybean in shape (as opposed to deep garnet reddish and tear drop shaped). This suggests that either these specimens are a different species to those described as *Alatina mordens* by Gershwin [1] or that the original description of *Alatina mordens* is inaccurate regarding this character. As specimens of *Alatina mordens* used for genetic comparative analysis by Bentlage et al. [29], [33] were provided by the first and third authors from this same collection trip, this discrepancy in identification may simply be from the original description [1]. Additionally, the original description of *Alatina moseri* [1] was made purely from preserved specimens and discrepancies in described features also exist when compared to specimens collected for this study. The predominant difference and a feature used to distinguish *Alatina moseri* is the presence of only 4 eyes in total [1], where as the specimens collected for these studies have 6 eyes per rhopalial niche. As type specimens of *Alatina moseri* do not match those used for this study, it again suggests that either these are a different species or that the original description is inaccurate as was solely based on preserved specimens and preservation can alter the appearance of certain features. Adult medusa from both collection sites most closely resembles the former species *Carybdea alata* and show identical morphological key taxonomic characteristics to each other. As *A. moseri* is the earliest named species of the two, for this paper we will refer to the animals in this study as *Alatina* cf. *moseri*.

Early developmental stages have been recently identified as important taxonomic indicators for both scyphozoans and cubozoans [13], [14], [16], [35], [36], [37] and could help shed light on the current species, family, order and even class debate. This study details the early life history of Australian and Hawaiian populations of *Alatina* cf. *moseri* comprehensively for the first time and compares to the description of *Carybdea alata* development from Puerto Rico [8] to see if there are developmental basis for species segregation. Additionally, these developmental observations will be compared to the current databases of cubozoan polyp life histories described and serve as a resource for ongoing research into cubozoan polyp ecology.

## Materials and Methods

### Ethics statement

No specific permissions were required for either of these locations as they were not taken from private or protected areas and cnidarians do not require permits for collection. Additionally, this species is not listed as protected or endangered.

### Collection of animals

Specimens from Waikiki Beach, Oahu, Hawaii

Specimens of *Alatina* cf. *moseri* (Plate S1, Fig. A) were collected from the shore at Waikiki, Hawaii (21°16′12.90″N, 157°49′24.22″W) between 2300 h and 0400 h on the 22^nd^ of October 2000, 10 days after the full moon. Individual animals that washed up onto the sand were collected by hand and placed individually into plastic click-seal bags with seawater. Over 80 individuals with milky white gonads and deemed to be in spawning condition (making the normally transparent bells cloudy) were retrieved.

Specimens from Osprey Reef, Northern Queensland, Australia

Large aggregations of the alatinid *Alatina* cf. *moseri* (Plate S1, Fig. B) had been noted regularly 8–10 days after the full moon at a site at Osprey Reef, North Queensland (13°52′55.17″S, 146°32′56.74″E) on a semi regular basis since 1999. Additionally at neighbouring reefs, mature individuals have been sighted with this same lunar periodicity. These additional sites include Raine Island in 2002 (11°35′30.19″S, 144°02′13.56″E), Holmes Reef in 2002 (16°27′13.68″S, 148°00′21.39″E), Miln Reef in 2004 (16°46′53.21″S, 146°15′53.81″E), Norman Reef in 2003 (16°25′36.51″S, 145°59′39.59″E), Agincourt Reef in 2003 (16°01′06.55″S, 145°51′01.40″E) and Steve's Bombie in 2007 (15°29′55.18″S, 145°48′05.90″E). Osprey Reef had been the site of five successful collection trips of mature *Alatina* cf. *moseri* individuals from 2000–2006 numbering between 30 to 300 animals per trip. This synchronicity allowed for predictable collection of mature specimens.

On November 15^th^, 2006, 10 days after full moon, specimens of *Alatina* cf. *moseri* were collected between 0000 h and 0230 h at Osprey Reef. To attract medusae for collection, high power underwater lights were set up off the diving platform of a research dive vessel anchored at the reef's edge. Collection of over 250 individual animals occurred, they were taken out of the water with dip nets and transferred into large collection bins filled with fresh seawater. Visual inspections of animals revealed approximately 60 percent of individuals were in spawning condition with milky white gonads evident.

### Fertilization

*Alatina* cf. *moseri* from Hawaii

The sexes of individual medusae were determined by light microscopy observation. For fertilization, approximately 0.5 mL of egg solution was extracted from each spawning female with a glass pipette and transferred into individual Petri dishes filled with clean, seawater. A collection of the water in which spawning males were kept was made, and from this “sperm mixture” a single drop was added to each of the egg solution dishes. Regular observations of cultures were made using a dissecting microscope with additional photographic documentation.

*Alatina* cf. *moseri* from Australia

Due to the mass storage of specimens in large buckets at the time of collection, individual fertilization of eggs was not required as the individuals spawned directly into the bucket. Multiple 650 mL plastic containers were filled with clean seawater and in each container approximately 1 mL of gamete mixture was added. The plastic containers were closed with lids and stored in a darkened room for transportation back to land. When back on land, observed planulae in these plastic containers were extracted and placed in new 650 ml containers with filtered seawater. Detailed observations concerning polyp formation were also documented via observations with a dissecting microscope and recorded with still images.

### Polyp cultures

The polyps of both *A.* cf. *moseri* populations were primarily cultured in the plastic containers and Petri dishes in which the planulae had settled. Additionally to the plastic containers, cultures of *Alatina* cf. *moseri* from Osprey Reef initially and later on cultures from Waikiki Beach were also cultured inside 30-L-BiOrb Fish Tanks® with filter systems. These were filled with fresh seawater and the whole content of plastic containers was suspended in the water column.

Cultures of both populations were held in a constant temperature aquarium room (24–26°C). Observations were made regularly over the following 18 months.

The polyps of both populations were fed with *Artemia* nauplii every second to third day from day 11 post fertilization onwards. Complete water changes of all the plastic containers occurred two hours post feed, with the large BiOrb® containers receiving half water changes every 3 to 6 months due to the filter systems.

In July 2007, 10 polyps from each of the *Alatina* cultures and 4 litres of original culture water for the adaptation period were transferred to the laboratory in the Zoological Institute in Hamburg, (Germany) to be continuously cultured by the second author for further detailed observations.

These polyps were kept at 25°C and fed two times a week with *Artemia* sp. nauplii. A complete water change with natural, filtered seawater followed about half an hour after feeding.

As the water contents and salinity might have differed from the sea water used in Hamburg, the polyps were first cultured in “Queensland water” which was diluted over the following three weeks with natural North Sea water for adaption. Detailed observations concerning polyp, metamorphosis and young medusa morphology and development were documented by photographs and measured as described by [14] [36].

For cultures in both countries, salinity levels were also kept at a constant range of 33–36 psu.

Abbreviations: MDD: mouth disc diameter; TBL: total body length; UD: umbrella diameter; UH: umbrella height

## Results

### Fertilization and polyp formation (Table S1)

Thorough observations of these two populations of polyps showed no difference between timelines of developmental stages or morphological features. As such all the data on development and anatomy have been combined from here on with any discrepancies noted.

For both populations the planulae were observed from 24 h to 48 h post gamete mixing in Petri dishes and at this stage showed the presence of “eye spots”. The planulae were then extracted and placed in clean plastic containers with seawater.

72 h post fertilization the planulae were observed on the bottom of the Petri dishes and had attained irregular shapes. Although predominantly round, 2 to 4 tentacle buds protruded from their circumference containing 2–4 small eurytele nematocysts in their tips. The tentacle buds continued to grow over the next 24 h.

On day 5, primary polyps were visible at the bottom of the container and moving in a creeping way. They had cone shaped bodies and 2–4 tentacles that were longer than the body length. They creep with one of the tentacles stretched out on the substrate like an antenna.

On day 6, approximately 50% of the primary polyps settled down on the bottom of the plastic containers and hypostomes were evident (Plate S1, Fig. D). At this stage, four 30-L-BiOrb® aquaria were filled with clean, filtered seawater and the plastic containers, with the settled polyps attached, were suspended in each of these BiOrbs®.

From day 10–13 a distinct shift in the morphology of the primary polyps was noted: they appeared to flatten against the substrate while their four tentacles were stretched out to the sides. The tentacles elongated and the euryteles in the tentacle tips were replaced by single stenoteles. At this stage polyps were fed for the first time on either finely sliced crayfish meat, ground up egg yolk, or the more successful diet of 1-day old *Artemia* nauplii (Plate S1, Fig. F). A feeding rate of approximately 30% of the individuals was noted on day 11. The polyp density was approximately 1 per 5 cm^2^. Polyps were subsequently fed with *Artemia* nauplii every second day.

From day 18 onwards, polyps increased in size and by day 21 approximately 40% of the polyps had developed six (Plate S1, Fig. E) or eight tentacles. These polyps with increased tentacle number started asexual reproduction (approximately 20% of polyps with buds at their calyx base, Plate S1, Fig. J). Asexual reproduction continued with polyps having as many as five buds at any time. These buds appeared to be in one of two forms and would either remain next to the base of the original ‘parent’ polyp after detachment, or undergo a creeping phase and relocate to another position. The creeping polyps from budding (Plate S1, Fig. K) did neither resemble the parent polyp nor the primary polyps during creeping phase (see creeping polyp description below).

Feeding regimes continued and asexual reproduction was apparent throughout the culture with exponential numbers of creeping polyps apparent over the sides of the Bio-Orb® tanks.

On day 29–31 the first signs of metamorphosis were apparent in all cultures with an observed change in polyp shape, a darkening of the polyp pigment and migration of the tentacles into four distinct clusters. At this time the hypostome of the polyp lengthened, as did the stalk. Continual and daily metamorphosis of polyps was observed and is noted in detail below.

PLATE S1 Medusae, polyps and asexual reproduction in *Alatina* cf. *moseri*. A: adult medusa, bell height ca. 100 mm – tentacles removed; B: adult medusa in the open water column (Australia); C: newly detached medusa; D: primary polyps (only few µm in height, no scale, picture was taken by camera through 400× microscope objective); E: young polyp (<20 days post fertilization); F: young polyp feeding on *Artemia* naupli; G: fully developed polyp (>21 days post fertilization), lateral view; H: fully developed polyp, oral view – note lips; I: hypostome, note lips (hypostome ca. 0.5 mm in length); J: budding polyp; K: creeping polyp (from bud); L: cyst, stage right after encystment, note the still visible mouth opening (MO); M: cyst, stage after a week, note the unstructured tissue inside the mucus shell.

### Polyp anatomy

The polyps of the two observed *Alatina* cf. *moseri* populations showed no distinct differences.

Polyps both populations were solitary (Plate S1, Fig. G). The bodies were divided into three parts, the hypostome, the calyx and a stalk region including a basal disc and a tiny periderm beaker enveloping the pedal region.

The polyps of both populations had a total body length ( = TBL) of up to 1.70 mm (TBL: 1.46–1.70, mean: 1.58 mm). The polyps of the Hawaiian population were only slightly larger with a total body length ( = TBL) of up to 1.70 mm (TBL: range: 1.46–1.70 mm) with a mean value of 1.58 mm (n = 25)) than the observed polyps of the Australian population (TBL: 1.43–1.63 mm, mean mm: 1.52 (n = 25)). The hypostome region included a single circlet of up to 19 tentacles (11–19, mean: 16, n = 25) in the Australian population. The tentacle numbers in the Hawaiian population ranged between 12–18 with a similar average of 16.The tentacles in both populations were solid, translucent, and bore a single stenotele in the knob-like tip. The calyces of both populations were shaped like a bottle, creamy-white with a tinge of orange when recently fed and about 60% of the TBL. A belt of nematocysts, mainly stenoteles and ovoid heterotrichous microbasic euryteles, was clearly visible around the lower calyx. The mouth disc diameter in both populations (MDD: Australian: 0.41–0.46, mean: 0.43; Hawaiian: 0.43–0.50; 0.48) was the widest body diameter and about 0.3fold of the calyx length.

The hypostome of polyps of both populations was four-lipped (Plate S1, Figs. H, I), balloon-shaped and approximately 14–15% of the TBL. It was not completely contractible into the body. The stalk, which was only slightly set off from the bottle shaped calyx, was short and colourless translucent.

Observations on feeding behaviour noted that the polyps of both populations showed a muscular ring at the level of the tentacular circlet which seemed to function as a water gate by constricting the calyx underneath the peristome until the opening to the gastrovascular cavity underneath the hypostome lumen was completely shut. In this way, the captured prey was placed into the hypostome and remained held there until the outer mouth opening was completely closed. After the opening had completely shut, this muscle ring relaxed and the opening to the stomach widened so that the prey could be transferred into the main gastric cavity.

### Asexual reproduction

Creeping polyps

There were no observed differences in the creeping polyps of either populations. Asexual proliferation in both populations occurred by lateral budding of polyps (origin: junction point of calyx and stalk, nematocyst developing zone), typically one to five buds per polyp at a time, depending on the feeding conditions. The creeping polyps showed 4 to 8 very short, still filiform tentacles around a cone-shaped hypostome and a high number of large stenotele nematocysts at the rear end of the body. Single stenoteles were located at the bases of the tentacles that migrated to the tentacle tips as soon as the polyps settled. The morphology of the creeping polyps differed a lot from the primary polyps by having a distinctly larger body size (TBL up to 2 mm) and by the “head region” which resembled a “daisy flower” resulting from the very short, nematocyst-lacking tentacles surrounding the mouth cone. These creeping polyps glided oral pole first, and, in contrary to the primary polyps, on their body midst with raised “head region” for 2 to 3 days over the substrate before they settled. After settlement they grew to a 9 to 12-tentacle stage before start budding themselves.

### Cysts

During extremely adverse conditions (drastic changes in temperature or salinity or absence of food for prolonged periods) the polyps of both populations encysted. To induce this cyst form, temperatures were lowered from 25°C to 19°C or salinity was increased to 42 psu or feeding was ceased for a period of four to five weeks. In this state polyps retracted their tentacles completely into their calyx and contracted the whole body into a ball-shape (Plate S1, Fig. L). The different parts of the body like tentacle tips, mouth opening and calyx fused into an indistinguishable white tissue ball, enclosed by a transparent and flexible mucus coat (Plate S1, Fig. M). The cysts of both populations stayed attached to the substrate. These survived for up to 3 months in these cyst forms during starvation events and both polyp populations subsequently regenerated within 3 days after feeding restart.

There were no obvious differences in the morphology of the endurance stages of populations from Australia or Hawaii, however, the longer conditions stayed unfavourable the more polyps died inside the cysts. The longest observed cyst state was by the Australian culture and this was for a twelve-month period before recovery was commenced.

PLATE S2 Metamorphosis in *Alatina* cf. *moseri* A: fully developed polyp; B: Stage 1: elongation of hypostome, calyx and stalk, tentacles cluster at four spots; C lateral view of stage 1 with tentacles being absorbed, D oral view close up of same animal as C: Stage 2: clustered tentacles fuse at base, note the red-violet pigmentation around hypostome and eye spots at the fused tentacles bases (D); E: Stage 3: medusa tentacles appear in space between rhopalia, F (same animal as E): note that this stage is still feeding on *Artemia* nauplii; G: Stage 4: nematocyst clusters appear on the developing exumbrella; H: Stage 5: after reabsorption of the remaining stalk tissue, the newly detached medusa free swimming.

### Metamorphosis (Plate S2)

Metamorphosis in *Alatina* cf. *moseri* polyps cultured in Germany, occurred only between the months of November and March, with and without temperature change. The difference with temperature change was that more polyps metamorphosed with raised temperature. The metamorphosis in Australian cultures occurred spontaneously since their original collection, however, not in the same mass numbers as was initially observed six weeks after initial fertilization took place. Both populations showed a complete metamorphosis of approximately 14 days from start of metamorphosis to medusa detachment. There were no morphological or time differences noted when comparing both populations. The stages of metamorphosis were documented photographically and described in detail (both populations combined) below:

Phase 1 (Plate S2, Fig. B): Elongation of calyx, stalk and especially hypostome, the hypostome in some cases being as long as the calyx and in all cases baseball bat-shaped. The tentacles formed clusters at 4 points around the peristome. Reddish violet pigmentation appeared at 4 spots between the tentacle at the base of the hypostome.

Phase 2 (Plate S2, Figs. C,D): The clustered tentacles fused at the bases, distal tentacle ends were reabsorbed and rhopalia developed at the fused tentacles bases. Statolith formation occurred initially while the tentacles were still reabsorbed. A horizontal ring groove appeared at the oral end of the calyx, dividing the transforming calyx into two parts: the upper part with already transformed medusoid tissue (plate-like cells) marked by reddish dark brown colour and the lower part with the not-yet transformed, unpigmented polypoid tissue (cylindrical cells) marked by white/pinkish colour (Plate S2, Fig. C). The reddish violet pigmentation sprat edgeways until a red-violet coloured pigment ring (Plate S2, Fig. D) was formed around the hypostome base.

Phase 3 (Plate S2, Figs. E, F): Between the rhopalia, 4 medusa tentacle buds appeared. A deep cleft around the hypostome marked the transformation of the polypoid hypostome into a medusoid manubrium. Single gastric filaments emerged in the interradii at the inner base of the manubrium. The red-violet pigmented region transformed into a velarium. The horizontal ring groove moved down towards the calyx base due to transformation of the remaining calyx tissue into medusoid tissue. Until this stage, the metamorphosing polyps were still catching and consuming prey (Plate S2, Fig. F).

Phase 4 (Plate S2, Fig. G): The medusoid tentacle buds grew out to very short, hollow and moniliform tentacles with four thick, yellowish brown coloured rolls of nematocyst batteries. The transformed calyx grew further in size, forming a slightly transparent umbrella and changing again colours from reddish dark brown to yellowish light brown. Small nematocyst clusters appeared, densely covering the developing exumbrella. The remaining stalk was reabsorbed.

Phase 5 (Plate S2, Fig. H): Pulsing of the medusae was initiated at least 48 h prior to detachment. At this stage no feeding behaviour in any of the newly metamorphosed polyps was observed. The medusa detached from the substrate while still a little remnant of the basal disc was visible at the apex of the umbrella. The basal disc was reabsorbed a few hours after detachment.

### Young medusa (Plate S2, Fig. H)

There were absolutely no differences found between the young medusae of these two populations. Newly detached medusae of both populations had tetraradial symmetry. The umbrella was spheroidally-pyramidal (Hawaiian and Australian population: umbrella height ( = UH): 1.2–1.6 mm, mean: 1.35 mm, umbrella diameter ( = UD): 1.1–1.5 mm, mean: 1.24 mm).

The bell warts of the medusae were small (0.06 mm in diameter), innumerable, round and densely covering the whole exumbrella and contained microbasic euryteles and spherical holotrichous isorhizas.

The manubria of the young medusae were short (0.24–0.3 mm, mean: 0.27; ≈20% of UH) and four-lipped. One gastric filament per quadrant was visible through the apex of the umbrella.

The medusae had 4 medusa tentacles that were much shorter than the length of the umbrella (approx. 16% of umbrella height). No additional medusa tentacles developed after detachment. Extended tentacles resembled short pearl strings because of the up to 4 thick, round, yellowish brown nematocyst batteries containing only ovoid heterotrichous microbasic euryteles. The young medusae were yellowish brown to yellowish olive in colour. Feeding of young medusae proved challenging with *Artemia* naupilli seeming insufficient to sustain them. The longest time span medusae (n = 6) were kept alive was for 29 days and were fed small pieces of raw *Acetes australis* three times a day. At their time of death, medusae ranged in size from 15 mm to 25 mm.

## Discussion

Several observations on the early life history of Carybdeida species have now been reported, however this is the first comprehensive description of the early life stages of two populations of the newly established genus *Alatina* [1].

### Fertilization & Polyp formation (Table S1)

Spawning aggregations have been previously reported in several species of Cubozoa [3], [4], [6], [7], [9], [12], [16], [17], [32], [38], [39], however, the investigations on *Alatina* sp. ( = *Carybdea alata* from Puerto Rico Arneson & Cutress [7], [8]), *Carybdea xaymacana* Conant, 1897 [32], [40], [41] , *Carybdea* sp. ( = *Carybdea marsupialis* from Puerto Rico, [94],[6] , *Carybdea rastoni* Haacke, 1887 [42] [5], *Copula sivickisi* (Stiasny, 1926) [43] [10], [12], [15] and *Tripedalia cystophora* Conant, 1897 [17], [20], [32], [41] have all shown internal fertilization occurs except in *Morbakka virulenta* (Kishinouye, 1910) where the gametes were released into the open water [16].

While it is unclear if internal fertilization was occurring in the observed populations of *Alatina* cf. *moseri* as was reported for Caribbean population of Alatina [7] there were obvious mass spawning events occurring for both these populations with eggs and sperm being released at time of collection.

The time spans of embryonic development stages are similar in most cubozoan species compared in Table S1. Fertilised eggs developed into blastulae within 24 h post fertilization (p.f.) which transformed into “eye spot” bearing planulae within 1–2 days p.f. and then settled approximately 2–3 days p.f. The only exceptions to this are the development of *Copula sivickisi* and *Morbakka virulenta*. In *C. sivickisi* the developmental time in the different stages were extended by up to 150% compared to the other described cubozoan species. This might be due to the unique reproductive strategy in this species where they produce an embryo strand and the embryos are not released from it until they have reached the planula stage [10], [12], [15]. In *Morbakka virulenta* the embryonic development was postponed for 21 days after reaching the blastula stage by forming a cyst as resting stage [16]. After the resting period the blastula transformed into an “eye spot”-lacking planula which stayed inside the egg shell until a 1-tentacled primary polyp hatched directly from the egg, using the shell as substrate [16].

In the original life cycle description of *Alatina sp. (Carybdea alata from Puerto Rico)* the settlement of the planulae was completed after 5–6 days p.f. [7] which is about twice as long as in our cultures of *Alatina* cf. *moseri*. Likewise, for the subsequent developmental rates, the stages were approximately one third slower in the *Alatina* sp. description compared to the developmental shown in our *Alatina* cf. *moseri* cultures. We suggest that the differences might be caused by the different culturing conditions, such as a lower rearing temperature, and not necessarily that these are different species as temperature and food supply can lead to differences in developmental rates [10],[44]. The planulae of *Alatina* cf. *moseri* settled on the bottom of PET-containers while the planulae of *Alatina* sp.settled on clean glass [7]. PET-substrate was used as this has previously been suggested as being the artificial substrate of choice for settlement of scyphozoan planulae [45], [46], [47].

### Polyps (Plate S1)

When comparing the polyps of both populations neither the measured sizes nor the morphology showed any significant difference. The general polyp morphology of the observed species conforms with that of other known cubopolyps in having (1) a bottle-shaped body with a clear structure and an aseptate gastric cavity, (2) solid, capitate tentacles with a stenotele nematocyst in their tips, and (3) a distinct stalk with a basal region covered by a thin periderm cuticle. The number of tentacles on polyps has been shown to differ between species [4], [6], [19], [48]. Polyps of *Alatina* sp. [7], [8] from Puerto Rico seem to be similar in structure and number with the polyps of *Alatina* cf. *moseri* when comparing the photos and descriptions and it is of note that the mean number of tentacles was identical. The polyps of *Carybdea* sp. (formerly described as *Carybdea marsupialis* by [4]), *Chironex fleckeri* [3]and *Carybdea morandinii* [14] do also show this general morphology in polyp tentacle structure [4], [6], [9], [48], [49]. The main exceptions to this polyp organisation are the polyps of *Tripedalia cystophora*, which have a bag-shaped body, fewer tentacles, and 20–40 heterotrichous euryteles in their tips instead of stenoteles [18], [19], [21], [22], [23], [35], [50], [51], [52], [53] and the polyps of *Morbakka virulenta* which have a tulip-shaped body which resembles more a scyphopolyp, and up to 17 tentacles with up to 30 nematocysts of two types (tri-rhopaloids and small spherical *p*-rhopaloids) located in their tips [16]. Additionally the polyps of *Carybdea morandinii* host zooxanthellae in their bodies [14], which were not found in either of our *Alatina* cf. *moseri* populations.

Polyps of *Alatina* cf. *moseri* show a muscular ring at the level of the tentacular circlet that seems to function in a similar way to the diaphragm found in *Carybdea morandinii* [14], [35], [53] by constricting the calyx underneath the peristome until the opening to the stomach underneath the hypostome lumen is completely shut. Like in *Carybdea morandinii* the muscle ring divides the gastric cavity horizontally into an antechamber and a main chamber but the diaphragm structure, which was found in *C. morandinii*, was not detected in the *Alatina* cf. *moseri*. These features can be observed with light microscopy while polyps are feeding.

A distinguishing characteristic of the observed *Alatina* cf. *moseri* populations is the four lips detected in the balloon shaped hypostome which resembles the club-shaped, four-lipped hypostomes of the scyphistoma of the Rhizostomida species [35], [37], [54], [55], [56], [57], [58], [59]. For Cubozoa this structure was previously unknown and was not noted in the *Alatina* sp. description by [7].

### Asexual reproduction

Creeping polyps

The budding process and the outer appearance of the creeping polyps resemble the description for other Carybdeida species [4], [6], [7], [8], [14], [35], [48], [53], [60] except the creeping polyps of *T. cystophora* [17], [18], [19], [21], [22], [61] which are very different from other species and resemble more the primary polyp stage than the creeping polyps and are also similar to those described for the chirodropid species *Chironex fleckeri* [3], [62]. An exception is again found in *Morbakka virulenta* which produces two-tentacled buds which do not develop head first but in a lateral position and which do not creep after detachment but swim with oral pole first near the bottom or the water surface [16].This kind of swimming behaviour was not observed in the *Alatina* cf. *moseri* .

Cysts

Encystment of cubopolyps were not originally described for *Alatina* sp [7] but were noted in *Tripedalia cystophora* [21], *Chironex fleckeri* [3] and *Carybdea morandinii* [14] during drastic salinity or temperature changes in culture waters. Encysting polyps were briefly described by Hartwick [3] as “a state that is probably equivalent to encystment where the polyps are highly contracted and non-responsive (*sensu* Werner [21])”. This state was further affirmed with the cyst formation being described again in *Tripedalia* and also noted in *Carybdea* sp. and *Carybdea morandinii* [35], [53]. The cysts of the two *Alatina* cf. *moseri* populations stuck to the substrate, similar to *Tripedalia*, and did not float around as seen in *Carybdea* sp. but as with those two species, the *Alatina* cf. *moseri* lacked structures like the anchor strings found in *Carybdea morandinii* [14], [35], [53].

For *Chironex fleckeri* it was noted that encysted polyps could last for at least two weeks in this form and would re-emerge to again feed if the salinity levels were returned to the original level [3]. Additionally, *Tripedalia* and *Carybdea* sp. cysts can survive for short periods (up to 14 days) of unfavourable conditions while the anchored cysts of *Carybdea morandinii* can survive for periods of up to 3 months [14], [53] . If the conditions did not return to normal after this time, the cysts died and macerated within 2–3 days. In comparison, the observed polyp cysts of the *Alatina* cf. *moseri* cultures seemed to be highly resilient, as they endured high salinities and starvation conditions for more than 12 months. The regeneration of *C. morandinii* polyps took 7 days after 14 days of encystment and up to 3 weeks after 1.5 months [14], [53] while the culture of Australian *Alatina* cf. *moseri* polyps regenerated back to their healthy feeding polyp state within 7 to 9 days after an encystment period of 12 months.

### Metamorphosis

Metamorphosis in both *Alatina* cf. *moseri* populations is complete and without any noted residuum (Metamorphosis Type 2 [13], [14], [35], [53]), as in other cubozoan life cycles [4], [6], [7], [8], [9], [18], [19], [21], [22], [23], [48], [50], [52] .

The whole process is very similar to the other cubozoan species, except for the highly extended hypostome, the capability of still feeding until a late stage (Phase 3) of the metamorphosis and the conspicuous, red-violet pigmentation of the future velarium region. This pigmentation was not mentioned for the metamorphosis of *Alatina* sp from Puerto Rico [7], [8].

The metamorphosis of the Hawaian and Australian *Alatina* cf. *moseri* populations results in a single 4-tentacled juvenile medusa as in *Alatina* sp, *Copula sivickisi*, *Carybdea* sp. (from Puerto Rico), *Tripedalia cystophora*, *Carybdea morandinii* and *Chironex fleckeri* [3], [4], [6], [7], [8], [9], [17], [18], [19], [21], [22], [23], [52], [53], [63].

A subsequent study on the same Australian population of *Alatina* (cited as *A latina* nr. *mordens*) has shown that while thermal and osmotic variations did not seem to vary the rate of metamorphosis in this species, the reduction of available food did [64].

### Newly detached medusae

As in *Alatina* sp [7], [8] in the exumbrella of the young medusae of the two *Alatina* cf. *moseri* populations, clusters of microbasic euryteles and spherical holotrichous isorhizas are found. This distribution of nematocyst types observed by us differs from *T. cystophora* and *C. marsupialis*, two species in which nematocyst clusters on the exumbrella mainly comprise atrichous and holotrichous isorhizas [21], [48] while in nettle warts on the exumbrella of *C. morandinii*, ovoid heterotrichous microbasic euryteles are predominant and isorhizas are rare. In the annular nematocyst batteries of the tentacles of *A* cf. *moseri* only ovoid heterotrichous microbasic euryteles are found like in *Alatina* sp from Puerto Rico, *Tripedalia cystophora* and *Carybdea* sp. [21], [48] while in the tentacles of *C. morandinii* several categories of nematocysts (holotrichous isorhizas, atrichous isorhizas, and single ovoid heterotrichous microbasic euryteles) are apparent [14]. The morphology of the newly detached medusae of *A*. cf. *moseri* are very similar to the medusae of *Alatina* sp described by [8] from Puerto Rico and the medusa drawn and described by Mayer [65] as *Charybdea aurifera* from the Tortugas.

### Notes on taxonomy

Early life history stages have recently been discussed as ancestral-relatedness-reflecting characters in Scyphozoa [35], [36], [37] and in Cubozoa [13], [14], [35] and also this study highlights that early life stages may be important indicators for taxonomical evaluations.

Not only does this study show that the early life stages from polyp to medusa of *Alatina* cf. *moseri* from both the Osprey Reef, Australia and Waikiki beach, Hawaii populations follow the identical developmental lines and show no significant differences in body sizes or morphology in both polyps and young and adult medusae. The morphological features of these populations are also remarkably similar to the stages described for the *Alatina* sp. (former *Carybdea alata*) from Puerto Rico, with noted variations potentially attributable to incomplete observations at that time. Additionally, it supports previous suggestions that within the *Alatina* genus there may be multiple species named which represent artificial taxonomic units [29], [33]. Previous DNA analysis has suggested that *Alatina mordens* and *Alatina moseri* to be the same species as there was no significant genetic divergences which corresponded to the geographical localities of these populations [29], [66]. Through observations of the sessile stage, development times, both adult and young medusa morphology as well as the structure of the cysts, this study supports the conclusion that the two populations of adult *Alatina* used in this study have displayed no evidence for species differentiation. The authors support further investigation into the other species comprising the newly formed genus *Alatina* in order to clarify the taxonomic issues that remain for this group.

## Supporting Information

Table S1 Time spans for embryology and primary polyp development in Cubozoa.
(DOCX)

Plate S1 Medusae, polyps and asexual reproduction in two *Alatina* cf *moseri* populations (Hawaii, Australia).
(DOCX)

Plate S2 Metamorphosis in *Alatina* cf *moseri* (Australian population).
(DOCX)
