# Supplementary material for: Early Life History of Alatina cf. moseri Populations from Australia and Hawaii with Implications for Taxonomy (Cubozoa: Carybdeida, Alatinidae)
Source: PLoS One. 2014 Jan 15;9(1):e84377. doi: 10.1371/journal.pone.0084377 (PMC3893091; doi:10.1371/journal.pone.0084377)
Supplement: Table S1 — Time spans for embryology and primary polyp development in Cubozoa. (DOCX) [file pone.0084377.s001.docx]

**Table S1 Time spans for embryology and primary polyp development in Cubozoa**

| **Event** | **Species** |  |  |  |  |  |  |  |  |
| --- | --- | --- | --- | --- | --- | --- | --- | --- | --- |
|  | ***Alatina* cf. *moseri* (both populations from Hawaii and Australia** | ***Alatina* sp. (formerly described as *Carybdea alata* from Puerto Rico)** | ***Carybdea* sp*.* (formerly described as *C. xaymacana*^1^ or *C. marsupialis*^3^ from Puerto Rico)** | ***Carybdea rastoni* (from Japan)** | ***Carybdea sivickisi* (from Japan)** | ***Carybdea sivickisi* (from Queensland)** | ***Tripedalia cystophora* (from Puerto Rico)** | ***Morbakka virulenta* (from Japan)** | ***Chironex fleckeri* (from Queensland)** |
| **Mating behaviour** | spawning aggregation | spawning aggregation | spawning aggregation | spawning aggregation | courtship | courtship | courtship | spawning aggregation | courtship |
| **Type of fertilization** | artificially external | internal | internal | internal | internal | internal | internal | external | artificially external / external |
| **Cite of planula development** | external | external | internal / external | internal | external in embryo strand | external in embryo strand | internal | external | external |
| **Embryological development (Age in hours (h) / days (d) / weeks (w), post fertilization (p.f.) or post planula release (p.p.r.)** | | | | | | |  |  |  |
| **Blastulae / embryo strand** | 8 h p.f. / no | 1 d p.f. / no | no data / no | no data / no | mean: 55 h p.f. (range: 44-66 h p.f.) / yes | no data / yes | no data | 4 h p.f. | no data / no |
| **Blastocyst** | - | - | - | - | - | - | - | 3 d p.f. |  |
| **Planulae** | 24 - 48 h p.f. | 2 d p.f. | 10 h p.f. | no data | 6 d p.f. | 5 d p.f. | 2 d p.f. | 24 d p.f. | 12 h p.f. |
| **Planulae settled** | 3 d p.f. | 6 d p.f. | 2 d p.f. | 2-3 d p.p.r. | 9.5 d p.f. | 8-10 d p.f. | 2-3 d p.f. | - | 2 d p.f. |
| **Primary polyp development** | |  |  |  |  |  |  |  |  |
| **1-4 Tentacle buds with 2-4 euryteles in tips** | 4 d p.f | 8 d p.f. | 4 d p.f. (no nematocysts in tentacle tips) | 4-5 d p.p.r. (no nematocysts in tentacle tips) | 14.5 d p.f. | 9-11 d p.f. | 4-6 d p.f. | 26 d p.f. | 3-5 d p.f. (no nematocysts in tentacle tips) |
| **2-4 Tentacles & hypostome** | 5-6 d p.f. | no data | 7-8 d p.f. | 4-5 d p.p.r. | 17.5 d p.f. | 14-16 d p.f. | 4-6 d p.f. | 39 d p.f. | 19 d p.f. |
| **Stenotele replaces euryteles** | 10 – 13 d p.f. | 31 d p.f. | no data | no data | no data | 14-16 d p.f. | - | - | 19 d p.f.(no euryteles) |
| **6-8 Tentacles** | 21 d p.f. | 31 d p.f. | no data | no data | no data | no data | 8-10 w p.f. | 9 w p.f. | no data |
| **Metamorphosis (start)** | 31 d p.f. | 68 d p.f. | no data | no data | no data | no data | 10-12 w (70-84 d) p.f. | no data | 58 d p.f. |
| **Reference** | present study | 7,8 | 32, 38, 4, 6 | 5 | 12,15 | 10 | 32,19,20 | 16 | 9,3 |

- : event does not occur in this species d: day(s) p.f.: post fertilization w: week(s) h: hour(s)
