# Supplementary material for: Early Life History of Alatina cf. moseri Populations from Australia and Hawaii with Implications for Taxonomy (Cubozoa: Carybdeida, Alatinidae)
Source: PLoS One. 2014 Jan 15;9(1):e84377. doi: 10.1371/journal.pone.0084377 (PMC3893091; doi:10.1371/journal.pone.0084377)
Supplement: Plate S1 — Medusae, polyps and asexual reproduction in two Alatina cf moseri populations (Hawaii, Australia). (DOCX) [file pone.0084377.s002.docx]

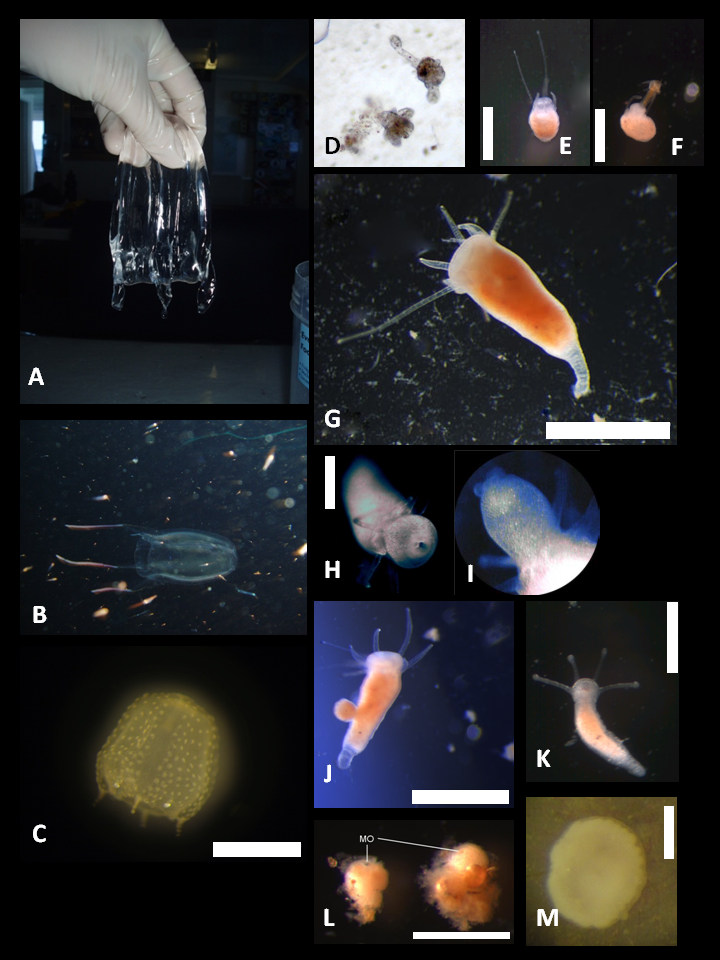


PLATE S1 Medusae, polyps and asexual reproduction in two *Alatina* nr *moseri* populations (Hawaii, Australia)

A: adult medusa from Hawaii, bell height ca. 100 mm – tentacles removed; B: adult medusa in the open water column (Australia); C: newly detached medusa (Hawaiian population); D: primary polyps (only few µm in height, no scale, picture was taken by camera through 400x microscope objective); E: young polyp (21 days post fertilization); F: young polyp feeding on *Artemia* nauplium; G: adult polyp (>21 days post fertilization), lateral view (Australian population); H: adult polyp, oral view – note lips (Australian population); I: hypostome, note lips (hypostome ca. 0.5 mm in length) (Hawaiian population); J: budding adult polyp (Hawaiian population); K: creeping polyp (bud); L: cyst, stage right after encystment, note the still visible mouth opening (MO); M: cyst, stage after a week, note the unstructured tissue inside the mucus shell.

Scale bars: A=100mm; C, G, J, L=1mm; E, F, H, K=0.5mm; M=0.25mm
