# Supplementary material for: Early Life History of Alatina cf. moseri Populations from Australia and Hawaii with Implications for Taxonomy (Cubozoa: Carybdeida, Alatinidae)
Source: PLoS One. 2014 Jan 15;9(1):e84377. doi: 10.1371/journal.pone.0084377 (PMC3893091; doi:10.1371/journal.pone.0084377)
Supplement: Plate S2 — Metamorphosis in Alatina cf moseri (Australian population). (DOCX) [file pone.0084377.s003.docx]

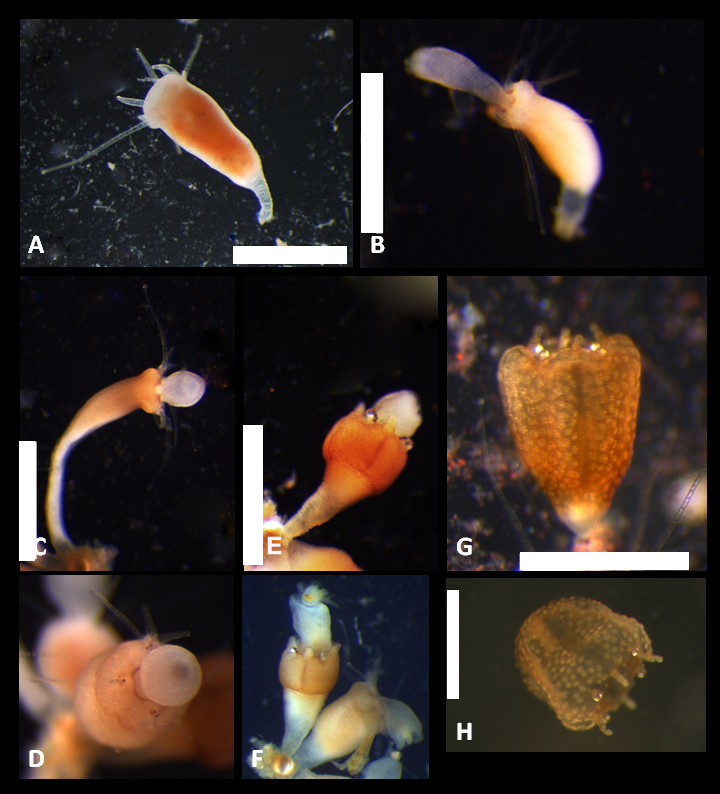


PLATE S2 Metamorphosis in *Alatina* cf *moseri* (Australian population).

A: adult polyp; B: Stage 1: elongation of hypostome, calyx and stalk, tentacles cluster at four spots; C (lateral view), D (oral view close up of same animal as C): Stage 2: clustered tentacles fuse at base, note the red-violet pigmentation around hypostome and eye spots at the fused tentacles bases (D); E: Stage 3: medusa tentacles appear in space between rhopalia, F (same animal as E): note that this stage is still feeding on *Artemia* nauplii; G: Stage 4: nematocyst clusters appear on the developing exumbrella; H: Stage 5: after reabsorption of the remaining stalk tissue, the newly detached medusa free swimming.

Scale bars: A, C, E, G, H=1mm; B=2mm
